# Visible‐Light‐Controlled Oxidation of Glucose using Titania‐Supported Silver Photocatalysts

**DOI:** 10.1002/cctc.201600775

**Published:** 2016-10-13

**Authors:** Luigi Da Vià, Carlo Recchi, Thomas E. Davies, Nicholas Greeves, Jose A. Lopez‐Sanchez

**Affiliations:** ^1^ Stephenson Institute for Renewable Energy, Chemistry DepartmentUniversity of LiverpoolCrown StreetL69 7ZDUK; ^2^ Chemistry DepartmentUniversity of LiverpoolCrown StreetL69 7ZDUK

**Keywords:** biomass, oxidation, photocatalysis, silver, supported catalysts

## Abstract

The visible‐light‐mediated photo‐catalytic selective valorisation of glucose using TiO_2_‐supported Ag nanoparticles is shown for the first time. The optimisation of the catalyst composition, substrate‐to‐catalyst ratio and reaction medium proved that a near total suppression of the mineralisation pathway could be achieved with a selectivity to partial oxidation products and small‐chain monosaccharides as high as 98 %. The primary products were determined to be gluconic acid, arabinose, erythrose, glyceraldehyde and formic acid. Under UVA light, the selectivity to organics decreases because of the production of CO_2_ from mineralisation. A reaction mechanism is proposed based on an α‐scission process combined with the Ruff degradation reaction, which explains the presence of the oxidation products, the smaller carbohydrates and formic acid. X‐ray photoelectron spectroscopy, UV/Vis spectroscopy and microscopy studies showed the presence of plasmonic 4 nm particles of silver that were oxidised to silver oxide over the course of the reaction, and recycling studies revealed that this was not detrimental to activity.

## Introduction

To date, photo‐catalysis has dealt typically with environmental remediation, sterilisation and decontamination of polluted water streams through the total oxidation or “mineralisation” of organic contaminants.^1^ Solar hydrogen production through water splitting and reforming is also a promising new technology for green fuel generation, although limitations in energy conversion efficiency, H_2_ production volumes, catalyst deactivation and H_2_ storage still fetter this emerging technology.^2^ Recent advances suggest that photo‐catalytic routes can perform selective oxidation,^3^ epoxidation, reduction, carbonylation and cyclisation reactions^4^ efficiently to offer a potentially inexpensive, green and chemically benign method for the functionalisation and transformation of chemicals. The selective photo‐catalytic oxidation of bio‐derived C_1_–C_4_ alcohols has been studied briefly, which indicates that high selectivities to the corresponding formates^5^ and aldehydes^6^ can be achieved if TiO_2_ is used as a catalyst. The conversion and decarboxylation of some organic acids using metal‐doped TiO_2_ 7 and Pt/TiO_2_ 8 catalysts have also been shown.

The harvesting of sunlight to drive chemical reactions for the production of high‐value chemicals from waste by‐products and renewable sources such as biomass is the natural next step in the creation of a green and sustainable chemical industry, but there are still challenges to overcome.^9^

Biomass comprises cellulose, hemicellulose and lignin, which are difficult substrates to work with because of their complex molecular structures and the large number of functional groups present on the carbon backbone. For this reason, research since the early 1970s has focussed on the conversion of smaller carbohydrates by photo‐catalytic routes. Glucose represents the ideal substrate; it is the most common and cheapest carbohydrate available in nature and it can be obtained from lignocellulosic waste biomass through the hydrolysis of its constituent polysaccharides (cellulose and hemicellulose). Glucose can be valorised into platform chemicals, such as glucaric acid, arabitol, levulinic acid and hydroxymethylfurfural, under relatively mild conditions with good yields.^10^ Early reviews surveyed the possible application of glucose oxidation products as chiral intermediates with potential application in the pharmaceutical industry and as precursors for vitamin C and other high‐value chemicals.^11^ Commercially, glucose is a precursor of gluconic acid, which is used in the pharmaceutical, food, health and textile industries.^12^ Glucose transformations are performed typically by fermentation and enzymatic routes but these often suffer from poor rates, low yields and the high cost of the enzyme used.^12^ An alternative selective route using a heterogeneous catalyst offers a potentially more robust pathway.

The selective catalytic oxidation of glucose continues to be of interest to researchers.^13^ As early as the 1940s, a number of scientists showed the efficiency of Pt and Pd‐based catalysts for the production of gluconic acid, which resulted in several patents.^14^ More recently, Au, Pd and Pt have been shown to be active and selective, which has led to a resurgence and further effort to make the reaction a more economically viable process.^13^, ^15^ However, typically, the reactions require the use of bubbling or pressurised O_2_ along with the constant addition of base to maintain the catalyst activity.13a, 15, 16

Along with the well‐established chemical conversion of sugars to platform chemicals, in recent years, academic attention has shifted towards the application of photo‐catalytic routes to obtain the same valuable chemicals using much less energy‐intensive processes and milder reaction conditions.

The photo‐conversion of carbohydrates to produce gluconic and glucaric acid using TiO_2_ catalysts under UV light has been investigated by Colmenares et al.^17^ More recently, detailed studies have been published by Chong et al.^18^ and Bellardita et al.^19^ in which several reaction mechanisms have been suggested to explain the reactivity of carbohydrates and the possible interactions with the several photoactive materials.^20^ Most recently, we have demonstrated that TiO_2_ can successfully convert glucose to higher value products under visible light through the formation of a ligand to metal charge transfer complex.^21^

The TiO_2_‐based materials tested in the studies cited above were tested under pure UVA irradiation or by using Xe lamps with no specific filters installed, which makes the comparison of the experimental results obtained difficult because of the variability of the reaction conditions.

Herein, we present a detailed study on the effect of both UVA and visible light upon the photo‐conversion of glucose using TiO_2_‐supported Ag nanoparticles and demonstrate for the first time that glucose can be converted to gluconic acid and other monosaccharides under visible light (*λ*>420 nm). The systematic analysis of several reaction parameters allowed the identification of a new reaction mechanism that comprises three reaction pathways by using HPLC with quadrupole time‐of‐flight mass spectrometry (Q‐TOF‐MS) to enable the determination of relevant and previously unaccounted for reaction products.

## Results and Discussion

### Photo‐catalysed glucose transformation under visible light

The decoration of the catalyst surface with metal nanoparticles (typically Ag, Au) extends the activity of the TiO_2_ support to the visible part of the electromagnetic spectrum because of the plasmonic effect. This interaction is responsible for the excitation of metal nanoparticles under visible light and the energy transfer from these nano‐antennas to the support and, subsequently, to the substrate.^22^

The 0.5–1.5 wt % Ag/TiO_2_ catalysts were prepared using a wet impregnation protocol and tested both under UVA and visible light. Initial tests performed using aqueous glucose solutions showed negligible catalytic activity, therefore, the MeCN/H_2_O (1:1 v/v) system studied by Colmenares et al.17d was investigated. Blank reactions were performed in the dark by using a Luzchem photoreactor (Figure S1) at 30 °C. None of the catalysts was active for glucose conversion under these conditions.

**Figure 1:**
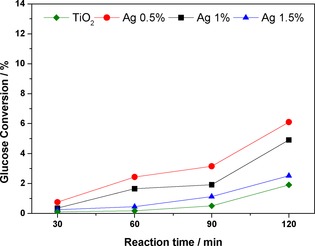
TOL glucose conversion under visible light over 120 min catalysed by Ag/TiO_2_ and bare TiO_2_. 50:50 v/v MeCN/H_2_O, 14 mg catalyst, 20 mm glucose stock solution.

The time‐on‐line (TOL) glucose conversion in which the bare TiO_2_ support is compared with those that bear nanoparticles is shown in Figure 1. The addition of the metal nanoparticles enhances the activity of the material towards glucose oxidation under visible light. However, this promotional effect is more pronounced if the lowest amount of Ag was used (0.5 wt %), which resulted in a threefold increased activity.

**Figure 2:**
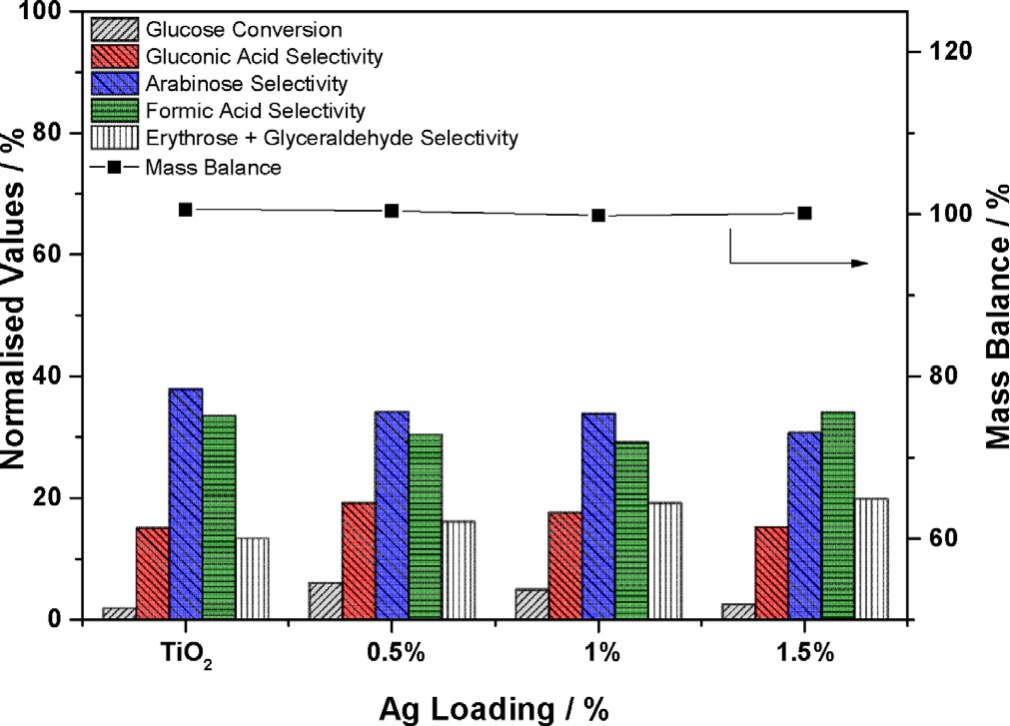
Glucose conversion, product distribution and mass balance data after 120 min of reaction under visible light. 50:50 v/v MeCN/H_2_O, 14 mg catalyst, 20 mm glucose stock solution.

The modest 2 % conversion recorded with the bare TiO_2_ after 120 min of irradiation increased to 6 % with the addition of low concentrations of Ag. The suppression of the mineralisation pathway to CO_2_ was essentially complete in all cases, as shown by the >99 % mass balance values (Figure 2).

However, by increasing the Ag loading to values higher than 0.5 % the glucose conversion decreased from 6 to 3 % for the 1.5 wt % Ag/TiO_2_ catalyst, which indicated that the availability of the TiO_2_ surface is a critical parameter as reported previously for Ag‐type systems by Grabowska et al.^23^ In a recent study, Fu et al.^24^ showed that the H_2_ generation from glucose reforming using Pt/TiO_2_ catalysts was related to the dispersion of the nanoparticles on the catalyst surface. Therefore, it appears that the best metal loading for glucose oxidation under the experimental conditions used in this study is 0.5 wt %. The reason for the catalytic enhancement if Ag nanoparticles are supported on a TiO_2_ semiconductor can be understood if we consider the Schottky barrier at the metal–support interface, which slows the electron–hole recombination, prolongs their lifetime and, subsequently, enhances the activity of the TiO_2_, as reported elsewhere.22a, 25

There is a correlation between the number of Schottky barriers and the catalytic efficiency of the material. If the metal nanoparticles are too close to each other, they act as electron–hole sinks or recombination centres, which thus reduces the availability of these species to participate in redox processes.

Finally, different metal loadings have a limited impact on the product distribution after 120 min of irradiation, and the selectivity towards the partial oxidation products is the same within experimental error. In all cases (which includes the bare TiO_2_) the main reaction products are arabinose (>35 %), formic acid (∼30 %) and gluconic acid (15–18 %) along with erythrose and glyceraldehyde (Figure 2). The product distribution values agree with previous results.17d, 18, 19

### Photo‐catalysed glucose transformation under UVA light

**Figure 3:**
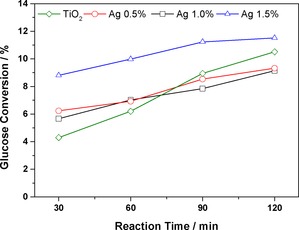
TOL glucose conversion under UVA light over 120 min catalysed by Ag/TiO_2_ and bare TiO_2_. 50:50 v/v MeCN/H_2_O, 14 mg catalyst, 20 mm glucose stock solution.

Glucose conversion under UVA light using bare TiO_2_ and the 0.5–1.5 wt % Ag/TiO_2_ catalysts is shown in Figure 3.

**Figure 4:**
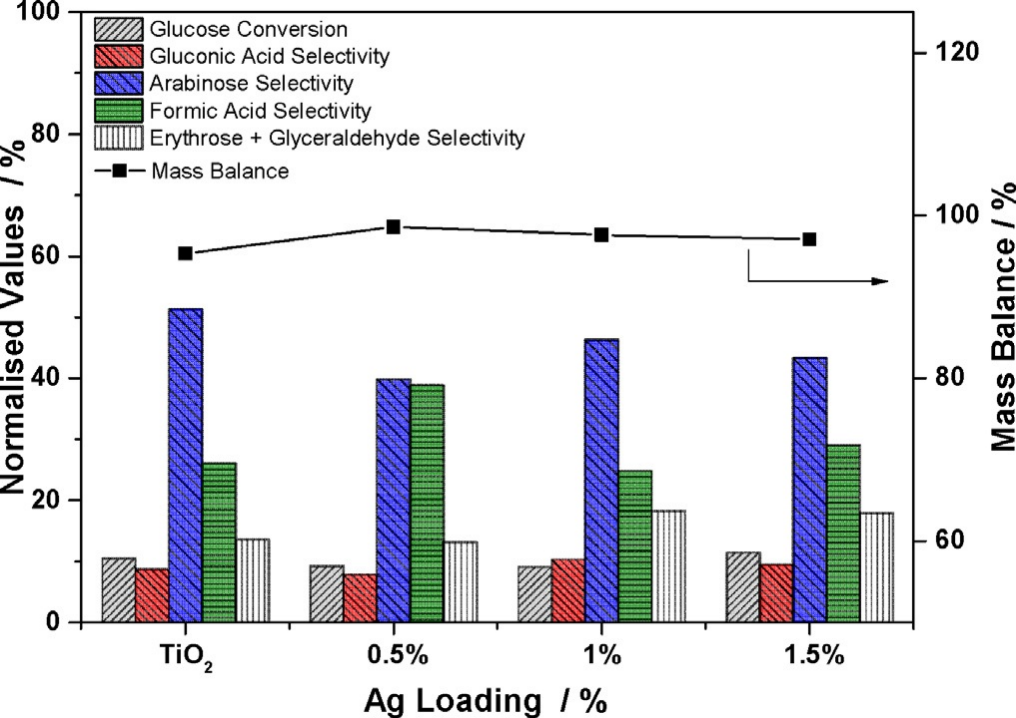
Glucose conversion, product distribution and mass balance data after 120 min of reaction under UVA light. 50:50 v/v MeCN/H_2_O, 14 mg catalyst, 20 mm glucose stock solution.

Under these conditions, the highest conversion (11.5 %) was achieved with the 1.5 wt % Ag/TiO_2_ catalyst versus 9 % for the bare TiO_2_. Under UVA irradiation, the presence of Ag nanoparticles has no beneficial effect on the activity of the materials for the selective oxidation process but does promote the mineralisation reaction for the production of CO_2_; the mass balance values get lower with an increased Ag loading from 0.5 to 1.5 wt % (Figure 4).

Unlike the previous case in which visible‐light irradiation was used, under UVA irradiation, both the support and the metal nanoparticles can be excited simultaneously, and the dominant reaction mechanism is the charge‐separation step in the TiO_2_ in which the photo‐generated electrons are transferred from the support to the metal nanoparticles.^26^

Also, the intra‐band transition of the electrons within the metal nanoparticle from the fully occupied d bands below the Fermi level to the half‐filled sp bands has to be considered. The activity of the Ag nanoparticles under these conditions is associated with the promotion of 4d electrons to 5sp orbitals.^27^ The holes left in the inner d orbital have a greater tendency to capture electrons than the outermost sp orbitals and, therefore, act similarly to the electron–hole couple generated in a semiconductor in which the electron vacancy in the d orbital acts as a hole.^28^ The energy required to promote these transitions is much higher, hence the necessity for UV irradiation. This mechanistic difference explains why under UVA irradiation the best‐performing material was that with the highest metal loading, which is different to our observations under visible light.

Although the combined excitation of the Ag nanoparticles and the TiO_2_ enhances the production of the radical species responsible for the photo‐activity of the material for example, OH^.^, reactive oxygen species (ROS) and h^+^, this results in unselective glucose conversion towards CO_2_. Additionally, we observed a shift in the product distribution values as arabinose is the primary reaction product with selectivity values above 40 % and gluconic acid values lower than 10 % in all cases (Figure 4).

## Reaction mechanism

**Figure 5:**
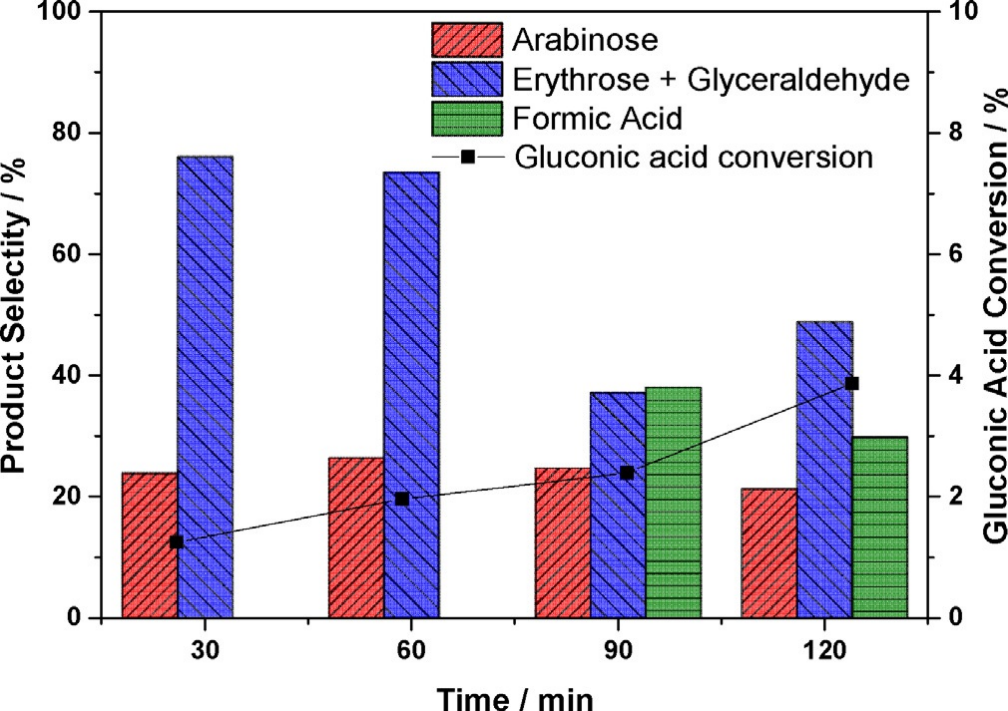
Gluconic acid conversion, product distribution and mass balance data after 120 min of reaction under visible light. 50:50 v/v MeCN/H_2_O, 14 mg catalyst, 20 mm gluconic acid stock solution.

The susceptibility of the reaction products and intermediates to further radical attack along with the difficulties in the quantification of complex mixtures of sugar isomers with oxidation and degradation products makes the full quantification of reaction products very challenging, that is, it is very difficult to achieve a 100 % mass balance. Therefore, it is extremely difficult to ascertain an adequate and comprehensive reaction mechanism for all the observed reactivity. Reaction mechanisms for the photo‐conversion of carbohydrates have been proposed before based on the observed reaction products and intermediates,17d, 18, 19 but the reaction pathways described are representative of the reaction set‐up used in each study, that is, the light source, source power, the solvent used and the photo‐catalyst. In some cases, the presence of particular molecules is neglected in the depiction of the reaction scheme. Stapley and BeMiller^29^ reviewed the decarboxylation of sugars and sugar acids to produce smaller‐chain carbohydrates. Specifically, they reviewed the so‐called Ruff degradation that involves the decarboxylation of aldonic acids by Fe^III^ and H_2_O_2_ in a Fenton‐like system to produce smaller carbohydrates. They also report that Ti^IV^ behaves similarly under the same reaction conditions, which explains the reaction mechanism in the absence of Fe^III^ species.^29^, ^30^ Based on this mechanism, we decided to investigate the behaviour of gluconic acid stock solutions under UVA and visible‐light irradiation to assess the resulting product distribution using the 0.5 wt % Ag/TiO_2_ catalyst (Figure 5 and Figure S5). The use of a 20 mm gluconic acid solution in the MeCN/H_2_O mixture produces a similar product distribution to that of the glucose substrate. With the metal‐supported catalyst, approximately 17 % gluconic acid could be detected after 30 min of UVA irradiation, whereas 120 min was necessary to obtain a similar selectivity if bare TiO_2_ was used (Figure S5). However, in the case of gluconic acid, significant amounts of formic acid were found, and its presence could not be linked directly to the Ruff mechanism as it involves the production of solely CO_2_.

Therefore, a more complex scheme that comprises multiple reaction pathways is needed to describe the system fully.

**Scheme 1:**
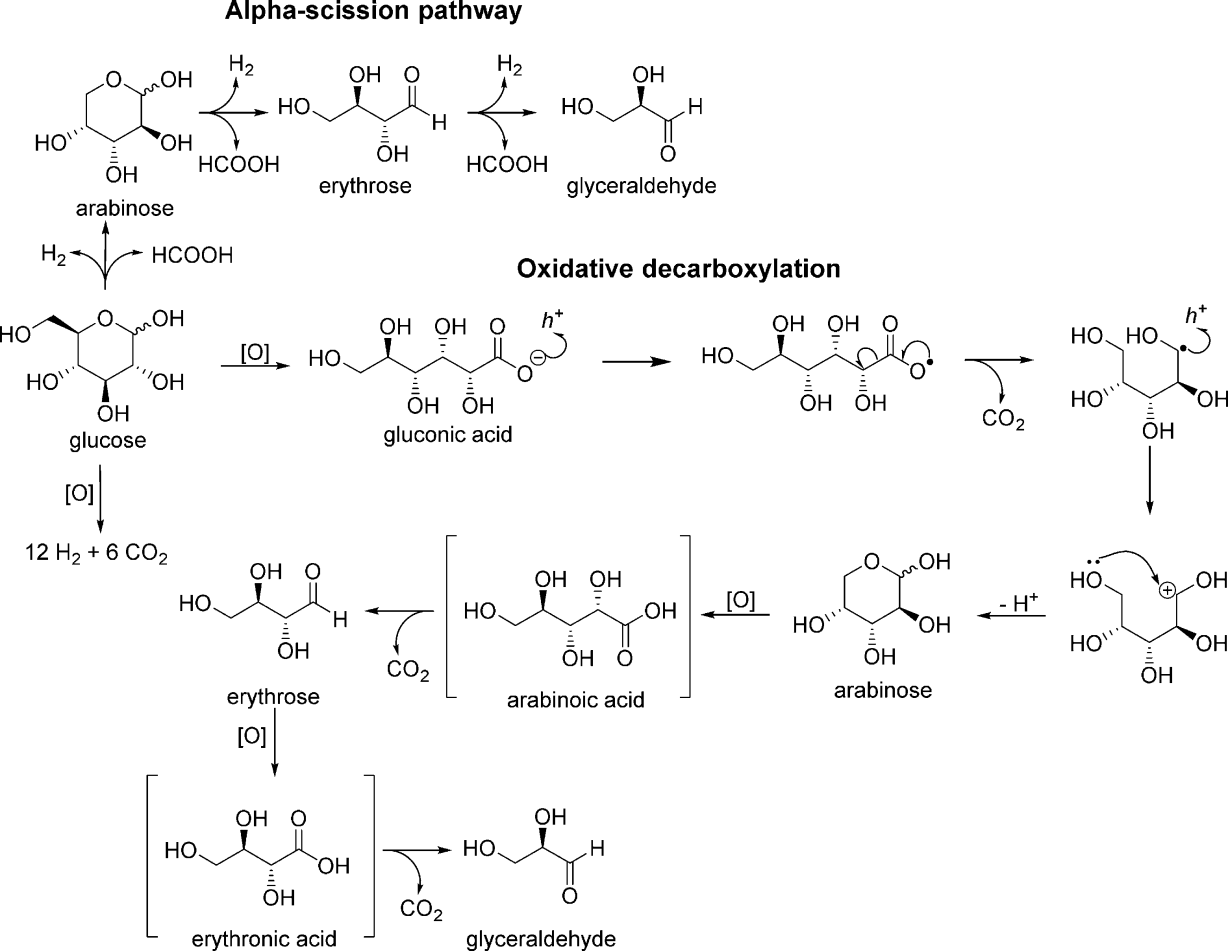
Proposed reaction mechanism for the photo‐catalytic conversion of glucose. This global pathway includes the α‐scission of sugars suggested by Chong et al.^18^ along with the oxidative decarboxylation mechanism typical of the Ruff degradation.^29^ This mechanism was found to be applicable to describe the reaction products obtained under visible and UVA light. The products shown in brackets could not be isolated from the reaction mixture.

The overall reaction mechanism depicted in Scheme 1 combines and explains the observations by Colmenares et al.17d and Chong et al.^18^ and, based on the findings of our experiments, also sees the inclusion of the Ruff degradation step. Our proposed mechanism agrees partly with the mechanism suggested by Chong et al.,^18^ whereby α‐scission generates the successive formation of shorter‐chain carbohydrates with the formation of equimolar hydrogen and formic acid. The H_2_ produced was determined qualitatively by using headspace GC analysis over 24 h reaction time (Figure S13), but because of the nature of the reaction it was not possible to determine the H_2_/CO_2_ ratio because of the mineralisation reaction that occurs in parallel with the α‐scission pathway, that is, not only is the production so low as to be near the detection limits of the analytical method employed but any H_2_ evolved from water splitting cannot be separated from the H_2_ from the mineralisation of the glucose. However, we could obtain high amounts of gluconic acid both under visible and UVA light (Figures 2 and 4), and an α‐scission mechanism alone does not explain the formation of glucose oxidation products (Scheme 1). We believe that photo‐catalytic oxidation reactions are responsible for the oxidation products observed and that the resulting acid products react further to decarboxylate as depicted in Scheme 1. From our data, it is apparent that the glucose is first oxidised to gluconic acid before it undergoes the α‐scission of the C_1_−C_2_ bond to allow the formation of arabinose and formic acid. The arabinose then undergoes subsequent repeat C−C cleavage to form erythrose and glyceraldehyde. Furthermore, the presence of CO_2_ as a product cannot be attributed solely to the mineralisation of formic acid as reported previously.

The photo‐catalytic conversion of gluconic acid produces a much‐simplified reaction profile (Scheme 1), whereby the only detected products correspond to consecutive α‐scission products in the order 1) arabinose, 2) erythrose, 3) glyceraldehyde and 4) formic acid. However, we were only able to detect formic acid after 90 min (Figure 5). It is clear that the carbohydrates are indeed formed from gluconic acid and that the reaction proceeds through the partial oxidation of glucose to gluconic acid and sequential decarboxylation. Notably, analysis of the products from the gluconic acid experiment by using MS revealed no glucaric acid present, which indicates that under these conditions the oxidation of gluconic acid to glucaric acid does not take place. Furthermore, no arabitol was observed in any of the reactions as had been reported previously.17d Although these reactions were conducted under different conditions, the presence of arabitol would be possible through the reduction of arabinose, but this would be unexpected in our case. Furthermore, the Q‐TOF‐MS analysis (Figures S6 and S7) shows the absence of arabitol for the two control reactions performed under UVA and visible light.

The role of the photo‐catalyst is to generate h^+^ during the photo‐catalytic process that act as anodes to oxidise the organic molecules absorbed on the surface of the catalyst: initially glucose to gluconic acid. As a result of the high oxidising potential, molecules adsorbed on the surface such as gluconic acid can undergo decarboxylation through a mechanism similar to a Ruff degradation (seen typically with Fe^III^ and H_2_O_2_), but performed here by Ti^IV^ and the photo‐generated radical species.^30^ This photo‐oxidative pathway does not result in the production of formic acid in each step, so it appears to be the dominant path during the early stages of the photo‐oxidation process (Figure 5). The appearance of formic acid at longer reaction times under visible light suggests that both the α‐scission and a Ruff‐type degradation mechanism take place simultaneously. However, under UVA irradiation, the presence of gluconic acid can be detected after 30 min of irradiation as highlighted previously (Figure S5). The production of the two acids (formic and gluconic) can only be explained in this case if the two reaction mechanisms (α‐scission and Ruff degradation) occur simultaneously. In this respect, it is evident how the metal nanoparticles promote the formation of gluconic acid as an intermediate in the glucose oxidative decarboxylation. The kinetic production of the acid intermediate is clearly faster than the α‐scission process as the 17 % selectivity observed at the beginning remains constant throughout the reaction. Further work on reaction conditions: [O_2_] control and the nature of the catalyst, reaction medium and irradiation source will potentially offer control with regard to which reaction pathways can be promoted or demoted and be the key to obtain selectivity and further insights into the mechanism. The effect of the substrate was analysed by using three glucose stock solutions with concentrations in the range of 2.8–20 mm. If the most dilute glucose solution was used (2.8 mm) the mineralisation pathway played a significant role in the glucose conversion with a mass balance value <90 % under visible light and as low as 82 % under UVA irradiation. If the substrate concentration was increased, the mass balance was significantly better with values greater than 95 % for the 20 mm stock solution. The increased substrate concentration did not affect the product distribution, and the relative ratio of the partial oxidation products remains within the experimental error (Figure S8). Therefore, it is clear how the surface coverage of the catalyst plays a pivotal role to determine the activity of the system and which reaction pathway will be more dominant.

### Catalysts characterisation and recycling studies

**Figure 6:**
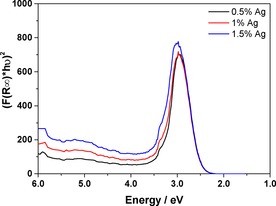
Solid‐state UV/Vis spectra of the fresh Ag/TiO_2_ catalysts. The peaks in the spectrum are caused by the plasmonic resonance of the Ag nanoparticles on the catalyst surface.

Solid‐state UV/Vis spectroscopy was used to assess the presence of the metal nanoparticles on the catalyst surface. We used a Tauc plot^31^ to evaluate the band gap of solid samples, and if the Kubelka–Munk function is used with the reflectance plotted against the wavelength energy^32^ it is feasible to isolate the presence of the metal nanoparticles from the support as shown in Figure 6.

**Figure 7:**
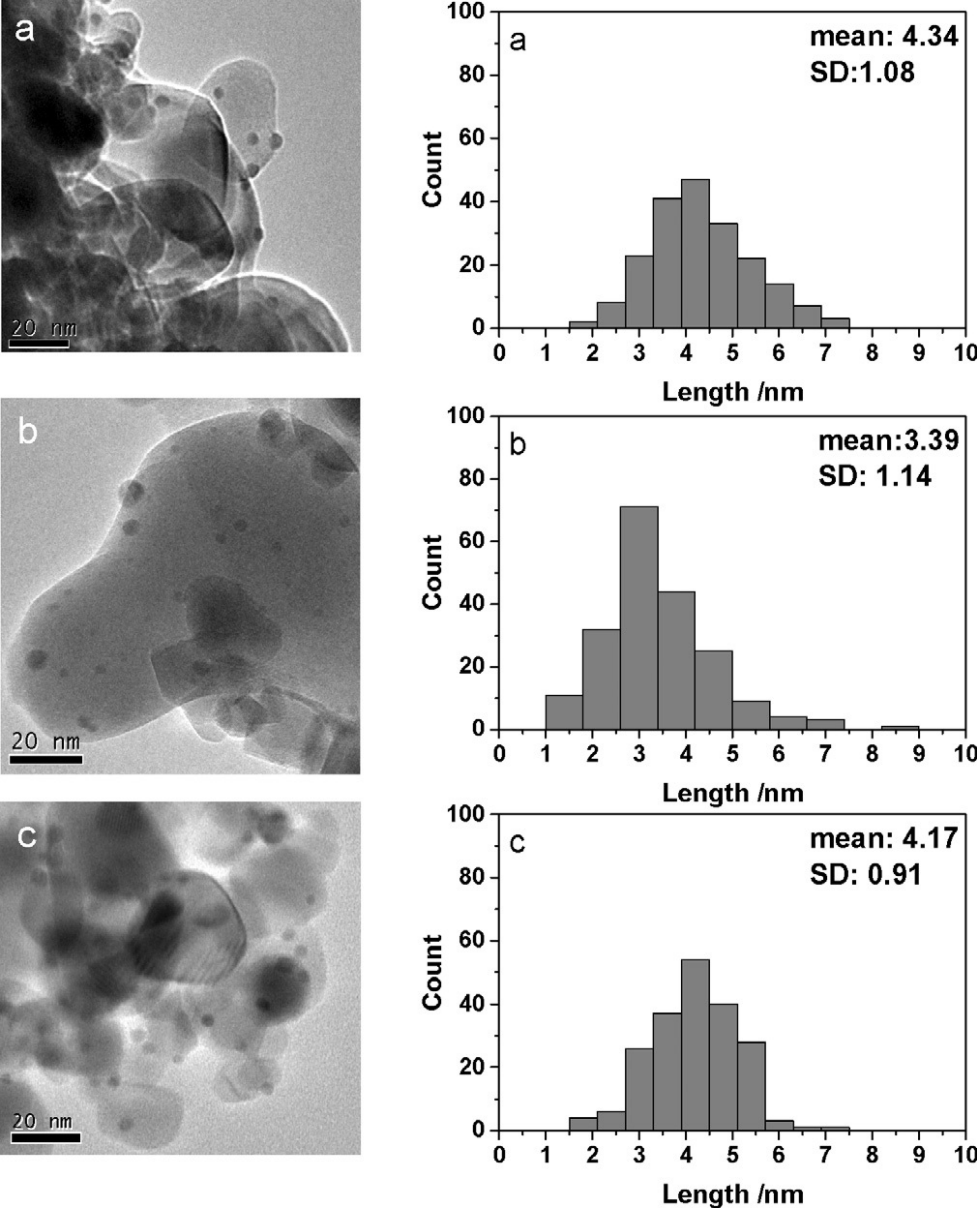
TEM images and particles size distributions of a) 0.5 wt % Ag/TiO_2_, b) 1.0 wt % Ag/TiO_2_ and c) 1.5 wt % Ag/TiO_2_.

**Figure 8:**
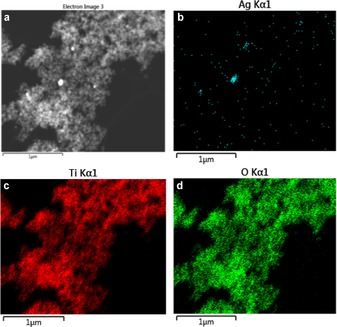
a) TEM image of recycled 1 wt % Ag/TiO_2_ under visible light; b, c, d) EDX elemental mapping of the recycled catalyst under visible light.

**Figure 9:**
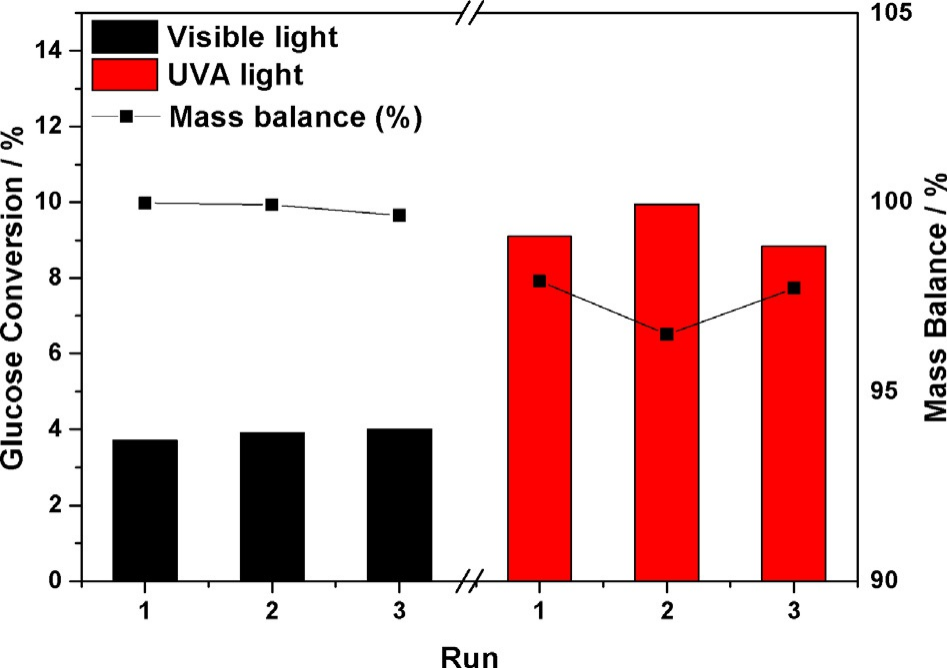
Catalyst recycling of 1 wt % Ag/TiO_2_ under visible light (black) and UVA light (red). The conversion and the mass balance values are taken from the samples after 120 min of irradiation.

The TEM images and particle size distribution determined from 300 particles for the 0.5–1.5 wt % Ag/TiO_2_ catalysts are shown in Figure 7. All catalysts have particles in the size range of 1–8 nm with a mean particle size of approximately 4 nm. Although the 1 % Ag/TiO_2_ had a mean average of 3.4 nm, recycling experiments indicate that an increase in particle size as a result of photo‐induced agglomeration has a minimal effect on the activity of the catalyst (Figures 8 and 9). Previous results show that plasmonic effects become negligible in nanoparticles smaller than 2 nm, and size differences between particles of <5 nm have a negligible effect on their absorbance wavelength.^33^ Elemental analysis showed the actual loading to be lower than the theoretical value in all cases, which was tolerable (Table 1).

**Table 1:** Elemental analysis and XPS data for the Ag/TiO_2_ catalysts and the recycled 1 % Ag/TiO_2_.

Catalyst	Ag content^[a]^	Ag content^[a]^	Ag 3d_5/2_ BE	Surface concentration^[b]^	Chemical state
	[wt %]	[at %]	[eV]	[at %]	
0.5 wt % Ag/TiO_2_	0.30 (±0.11)	0.06	368.1	0.01	Ag/Ag_2_O
1.0 wt % Ag/TiO_2_	0.78 (±0.15)	0.18	368.0	0.08	Ag/Ag_2_O
1.5 wt % Ag/TiO_2_	1.34 (±0.15)	0.30	367.7	0.89	Ag_2_O
Recycle 1^[c]^	–	–	367.4	0.05	AgO
Recycle 2 Xe	0.67 (±0.15)	0.15	367.3	0.03	AgO
Recycle 2 UV	0.75 (±0.16)	0.15	–	–	–

[a] Determined by using EDX over three areas. [b] Determined by using XPS. [c] 1.0 wt % Ag/TiO_2_ after three recycles; 2 h under visible light.

**Figure 10:**
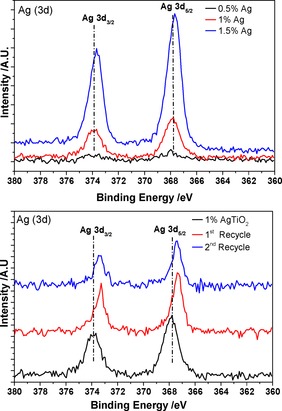
XPS analysis of the fresh Ag/TiO_2_ catalysts (top) and 1 % Ag/TiO_2_ after multiple recycling tests (bottom).

The chemical environment and valence state were determined by using X‐ray photoelectron spectroscopy (XPS), and the Ag 3d_3/2_ and 3d_5/2_ peaks are shown in Figure 10. The binding energies (BEs) for Ag, Ag_2_O and AgO are very close within 367.3–368.4 eV, but there is a clear shift in binding energy from 368.1 to 367.7 eV with increased Ag loading indicative of the formation of Ag^+1^ species. The ease of oxidation of Ag nanoparticles under air is not without precedent.^34^ The presence of the oxidised Ag species cannot be linked directly to the nature of the reaction considered and did not hinder the photo‐activity of the material, and Ag_2_O species on TiO_2_ supports have been shown previously to be active under visible irradiation.^35^

The presence of both metallic Ag and the oxides in all the catalysts cannot be discounted as Ag^0^ was evident from the TEM analysis of 0.5 wt % Ag/TiO_2_ (Figure S9) in which the interplanar distance of 0.24 nm for the particles can be attributed to the preferential exposure of the 1 1 1 plane (ICDD 01‐071‐3672).

Representative solid UV/Vis spectra of 1 wt % Ag/TiO_2_ (Figure S10) display a redshift in the plasmonic‐resonance peak after multiple re‐uses both under visible and UVA light. This slight shift from *λ*=383 to 420 nm is caused by oxide formation and changes in the nanoparticles morphology after multiple re‐uses.35d XPS of the catalyst after multiple re‐uses showed that the binding energy shifts to 367.3 eV indicative of the presence of Ag^2+^ species, in this case, AgO; this has been reported previously for similar systems.35d, 36

The signal attenuation corresponds to an apparent decrease in the surface concentration from 0.08 to 0.03 at % (Table 1). However, the energy‐dispersive X‐ray spectroscopy (EDX) microanalysis shows that the bulk concentration remains constant at approximately 0.1 at % (a value in good agreement with the 0.08 at % obtained by using XPS), which demonstrates that there is no leaching of the metal and that the lower surface concentration is caused by particle sintering.

Further investigation by using EDX mapping showed that the lower surface concentration determined by using XPS is a result of light‐induced particle agglomeration as large particles >200 nm can be seen throughout the titania matrix (Figure 8).

Interestingly, the same effect was not observed for the catalysts recycled under UVA light (data not shown). Ag nanoparticles are known to show photo‐chromic behaviour if they are exposed to different light sources, which involves morphological changes of the supported metal nanoparticles because of the interaction of the incident light as reported elsewhere.^37^ Upon illumination of the supported nanoparticles, it is possible to influence their shape to obtain smaller satellite metallic structures from bigger particles.^38^ Additionally, the accumulation of the well‐dispersed nanoparticles into larger agglomerates was also observed. This effect can be explained by the super‐heating of the metal nanoparticles upon irradiation. Huang et al.^39^ showed recently that Ag nanoparticles can be super‐heated with femto‐laser pulses and that the strong electric fields cause the agglomeration of the particles into larger structures. Even in our case after exposure to the various light sources, the powders turned to a brownish grey colour as reported by Naoi et al.^40^ This colour change is caused by changes in the morphology and size of the nanoparticles as well as in the refractive index of the support. The glucose adsorption mechanism and the colour change associated with the formation of the glucose‐TiO_2_ complex reported by Kim et al.^41^ show how the refractive index of the TiO_2_ support is affected by the support–substrate interaction as shown by the FTIR spectroscopic analysis performed on the 0.5 wt % Ag/TiO_2_ catalyst after multiple re‐uses under visible and UVA light (Figure S11).

However, despite the morphological changes of the catalysts after multiple re‐uses, the activity and product distribution values remained unaffected. The data presented in Figure 9 show that the glucose conversion under both UVA and visible light for 1 wt % Ag/TiO_2_ remains at approximately 10 and 4 %, respectively, and retains the same mass balance and product distribution values after three runs (Figure S12).

## Conclusions

Here, for the first time, we have shown how visible light can be used to transform the renewable feedstock glucose to higher‐value organics such as gluconic acid, arabinose and formic acid using Ag/TiO_2_ catalysts. The promotion of TiO_2_ using plasmonic Ag nanoparticles resulted in enhanced conversion and high selectivity (>98 %) to the desired products with a near total suppression of the mineralisation pathway. The catalyst was re‐usable and showed no loss in activity or changes in the product distribution. We used TEM analysis to reveal how the nanoparticles are unstable under reaction conditions but this was not detrimental to activity. In addition to Ag, we anticipate that other plasmonic nanoparticles, such as Au, Cu and their alloys, could promote this reaction similarly to offer a new avenue to control the selective photo‐catalytic upgrading of bio‐derived polyols and saccharides using visible light.

## Experimental Section

### Ag/TiO_2_ synthesis

AgNO_3_ solutions were prepared by solubilising the appropriate amount of the salt in H_2_O. The support (TiO_2_, P25 Evonik) was suspended in 4 mL of H_2_O in a vial under magnetic stirring. The appropriate volume of the metal solution was then added, and the solution was left to evaporate under constant stirring at 80 °C until it became a paste. The supported catalysts were dried overnight under vacuum at 110 °C. The final dried catalysts were calcined under static air at 400 °C for 3 h at 2 °C min^−1^.

### Recycling of Ag/TiO_2_

The recycling of the Ag catalyst under visible and UV radiation was performed following a pyramidal scheme: three reactions were run for 2 h under the same experimental conditions using 14 mg of catalyst. The recovered catalyst was centrifuged, and the supernatant was removed. The catalysts were then washed with H_2_O and ethanol three times to remove any organics adsorbed on the catalyst. The washed powders were dried overnight at 50 °C and then ground. The recovered catalyst was used in the second run for two reactions and, after the reaction, it was treated following the procedure described above. Finally, for the third run, only one reaction was analysed. The values provided for the conversions and the product selectivity for the first and the second run are the average of the results obtained for each of the reactions.

### Catalyst testing

The reactions were performed in 16 mL glass vials with magnetic stirring. Typically, solutions at different glucose concentrations (2.8, 10 and 20 mm) were prepared by solubilising the substrate in a 50:50 v/v MeCN/H_2_O solution. Subsequently, 14 mg of catalyst was added to the solution. The reactions were performed for 2 h, and samples were taken every 30 min for the first 2 h and then hourly until the end of the reaction. The photo‐catalytic reactions were performed by using two different systems: a Luzchem Photoreactor (Mod. LZC‐4, Luzchem Research Inc. ON, CAN) equipped with 14 lamps (8 W each) for a total power of 112 W (Figures S1 and S2). The temperature was kept constant at 30 °C for all the reactions. The second system was a 300 W Xe Oriel Lamp (Mod. 6259, Newport, UK). The lamp was equipped with several filters to isolate just the visible part of the electromagnetic spectrum; a liquid filter to remove the IR region and a coloured glass filter with a cut‐off value of 420 nm (Mod.FSQ‐GG420, Newport, UK; Figures S3 and S4). The reaction mixture was stirred magnetically at a constant distance from the light source.

### Product analysis

The standard solution and the reaction products were analysed by using a 1200 HPLC Agilent (Agilent, USA) system equipped with an inline degasser, a quaternary pump, an autosampler and a column switch. The selected detectors were a photodiode array detector (DAD) and a refractive index detector (RID). The analytical column was an Aminex HPX‐87H (300 mm×7.8 mm), 9 μm particle size (Bio‐Rad CA, USA) column kept at 65 °C with 0.025 m H_2_SO_4_ as eluent with a flow rate of 0.65 mL min^−1^. Before analysis, the samples were centrifuged at 13 400 rpm for 1 min to remove any suspended particles. The glucose and the reaction products were determined using commercially available standards. The accurate mass of the oxidised products obtained from glucose was analysed by using an Agilent 6510 LC–Q‐TOF‐MS system and interpreted by using Agilent MassHunter Workstation Software (Version B.06.00). The column used for the MS analysis was a Varian MetaCarb 67H (300 mm×6.5 mm; Agilent, USA) kept at 65 °C using a 0.1 % w/w formic acid aqueous solution at a flow rate of 0.8 mL min^−1^. The Q‐TOF‐MS was operated in positive ESI mode.

### Catalyst characterisation

TEM was performed by using a JEOL 2100 instrument (Jeol Ltd, JPN) operated at 200 kV. Samples were prepared by dispersion in methanol with sonication and deposited on a 300 mesh holey carbon film.

XPS analysis was performed by using a Thermo K‐Alpha (Thermo Scientific, East Grinstead, UK) with a micro‐focused mono‐chromatic AlK_α_ source (1486.6 eV, 12 kV, 3 mA, 36 W) with a spot size of 400×800 μm. The data acquired were obtained from the analysis of three positions per sample with a general 30 scan survey and a 10 scan survey for the high‐resolution regions. The raw data were corrected by using the C 1s binding energy at 284.7 eV. The recorded spectra were fitted with least squares to produce Gaussian–Lorentzian functions after the subtraction of background noise.

Solid‐state UV/Vis spectroscopy was performed by using a UV‐2550 Shimadzu spectrophotometer equipped with an ISR‐2200 integrating sphere (Shimadzu Corp, JP) in the range of 200–800 nm with a 0.5 nm sampling interval and a 5 nm slit using BaSO_4_ as reference. The reflectance data were used to calculate the Kubelka–Munk function using the absolute reflectance (*R*_∞_) to determine the plasmon resonance of the supported Ag nanoparticles.

ATR‐FTIR spectra were recorded by using an HTS‐XT Bruker Tensor 27 (Bruker, USA) in the range 6000–400 cm^−1^ (resolution 4 cm^−1^), and 32 interferograms were recorded for each sample.

The EDX and SEM analysis of the samples was performed by using a Hitachi S‐4800 field‐emission microscope equipped with an Oxford Instruments Inca Energy EDX detector. The electron accelerating voltage was 30 kV with a probe current of 20 μA. Samples were analysed uncoated and the EDX measurements represent the average of a minimum of three points over the material.

## Supporting information

As a service to our authors and readers, this journal provides supporting information supplied by the authors. Such materials are peer reviewed and may be re‐organized for online delivery, but are not copy‐edited or typeset. Technical support issues arising from supporting information (other than missing files) should be addressed to the authors.

Supplementary
