# Supplementary material for: Visible‐Light‐Controlled Oxidation of Glucose using Titania‐Supported Silver Photocatalysts
Source: ChemCatChem. 2016 Oct 13;8(22):3475–83. doi: 10.1002/cctc.201600775 (PMC5396341; doi:10.1002/cctc.201600775)
Supplement: Supplementary file 1 — Supplementary [file CCTC-8-3475-s001.pdf]

Heterogeneous & Homogeneous & Bio- & Nano-
CHEMCATCHEM
CATALYSIS

Supporting Information

Visible-Light-Controlled Oxidation of Glucose using Titania-Supported Silver Photocatalysts

Luigi Da Vià,[a] Carlo Recchi,[a] Thomas E. Davies,[a] Nicholas Greeves,[b] and Jose A. Lopez-Sanchez*[a]

cctc_201600775_sm_miscellaneous_information.pdf

Supplementary Information

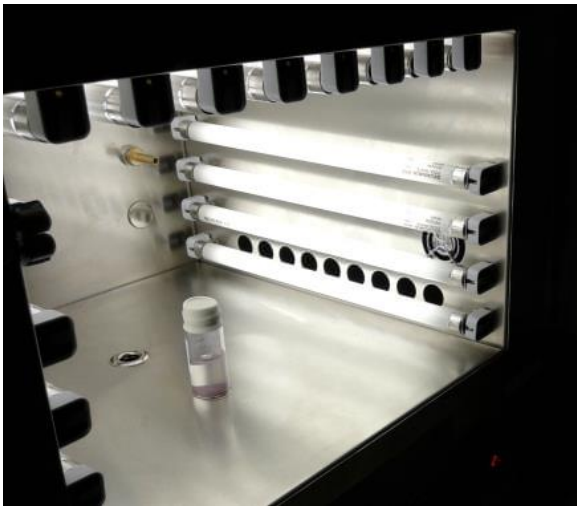

Figure S.1 Picture of the Luzchem Photoreactor equipped with the 14 8W lamps. The photoreactor is equipped with a magnetic stirrer and a programmable temperature controller from ambient temperature to 60°C.

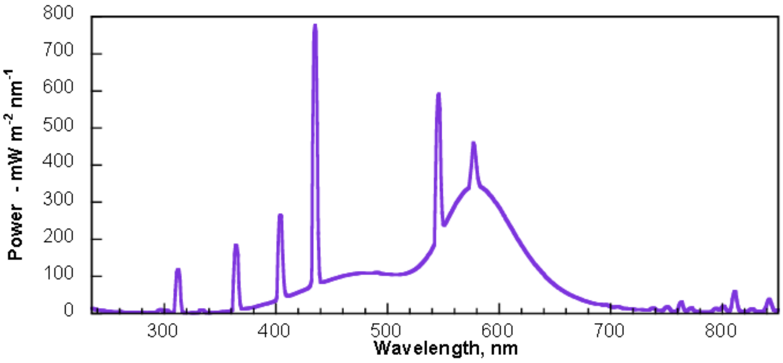

Region	Range, nm	Dose mWm^{-2}	% energy
UVA	316-400	1754	3.34
UVB	281-315	670.0	1.28
UVC	235-280	269.0	0.51
Visible	401-700	47837	91.16
NIR	701-850	1945	3.71

Figure S.2 Emission spectrum of the VIS Luzchem lamps measured in the range 235-850nm at 25°C. Resolved peaks at 312, 365, 405, 438, 546, 576, 581(broad) and 811nm. The table shows the energy distribution at the target expressed as a percentage of the total energy in the monitored range. Data from Luzchem Exposure Standards (www.luzchem.com).

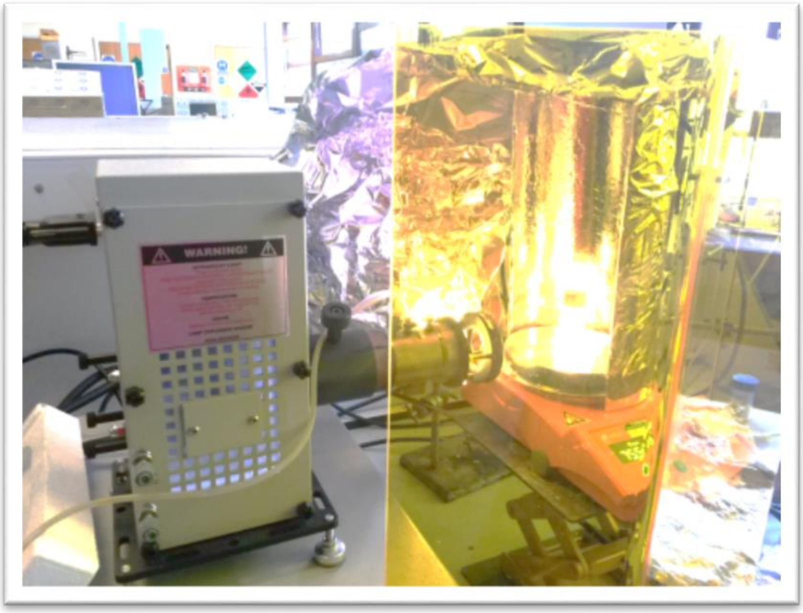

Figure S.3 Picture of the 300 W Xenon lamp used in the study. The lamp was equipped with liquid IR and visible light filters.

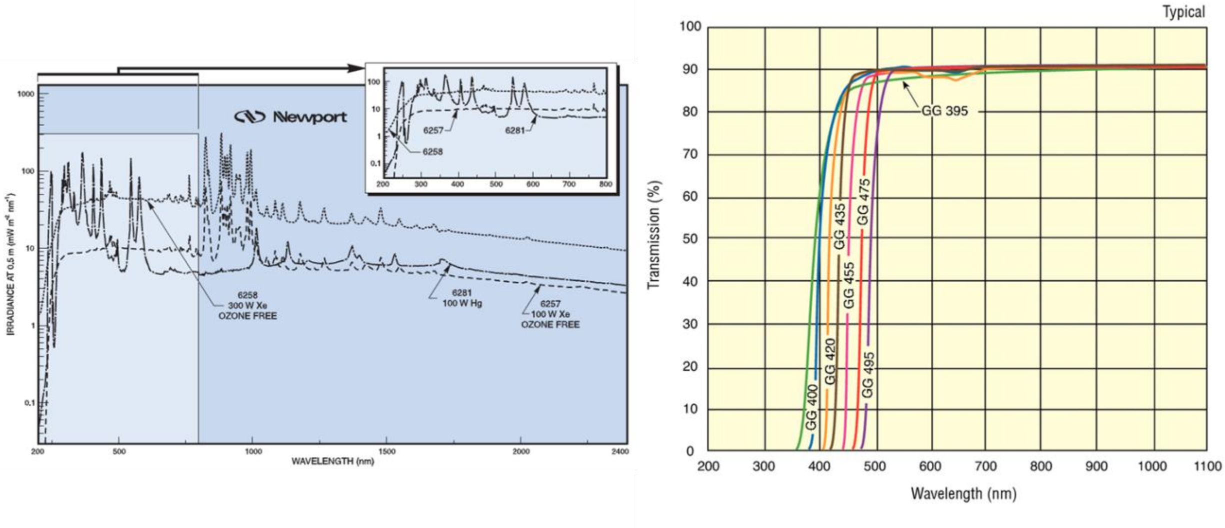

Figure S.4 In this picture are reported the emission spectrum for the 300W Xe lamp (on the left) and the transmission spectrum for various visible filters. In particular, the filter GG 420 used in this set of experiments, displays a 50% transmission value at 420 nm and a 90 % above ca.460nm in the visible light region.

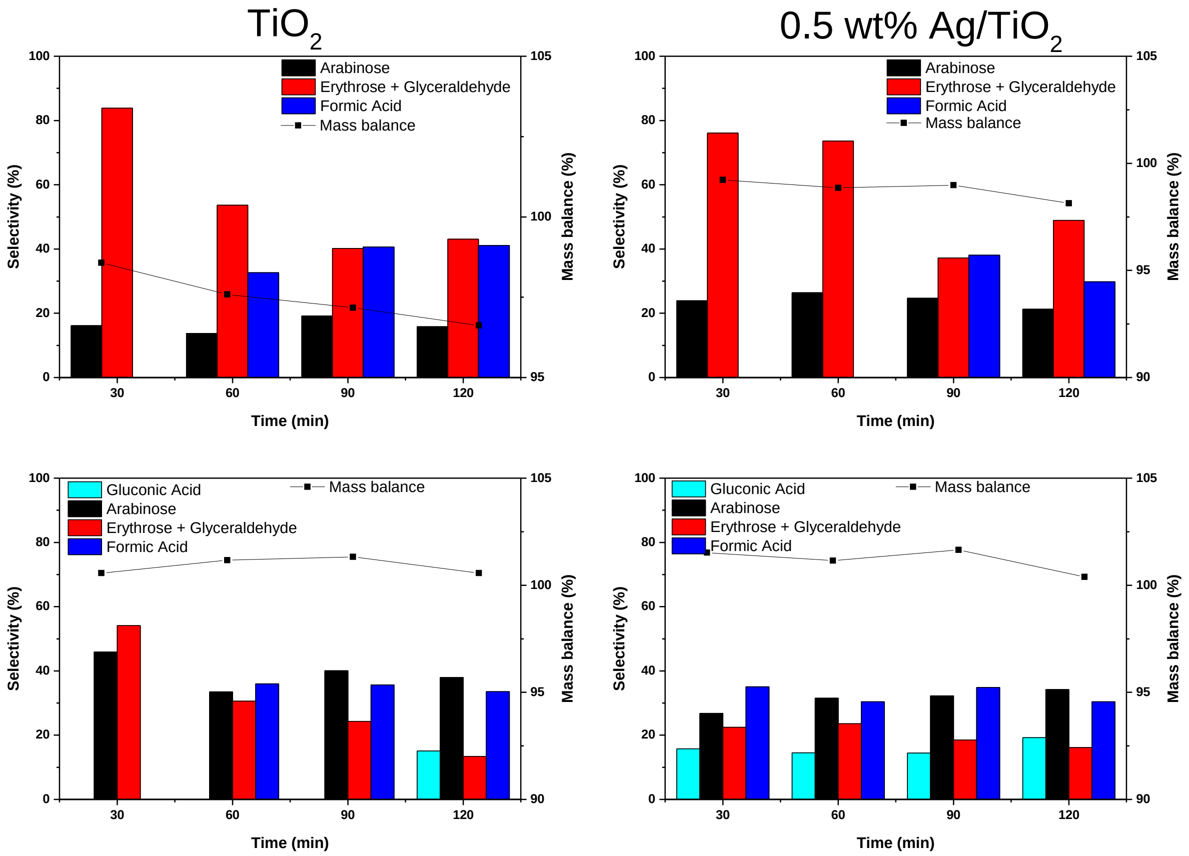

Figure S.5 TOL of the selectivity and mass balance data for the TiO_2 and 0.5% $AgTiO_2$ using gluconic acid (top) and glucose as a substrate (bottom). UVA light, 50/50 v/v $MeCN/H_2O$, 14mg catalyst, 20mM gluconic acid and glucose stock solutions.

QTOF analysis

The total ion chromatograms (TIC) of the samples obtained from the reaction with the 10mM glucose solution after 4 hours are reported in Figure S.5. In Figure S.6 are reported the TIC for the same reaction samples along with the TIC relative to the glucose, arabinose, and arabitol standards. It can be seen how the retention time of the arabinose matches the peak observed in the samples, presence confirmed by the $[M\text{-}Na]^+$ adduct.

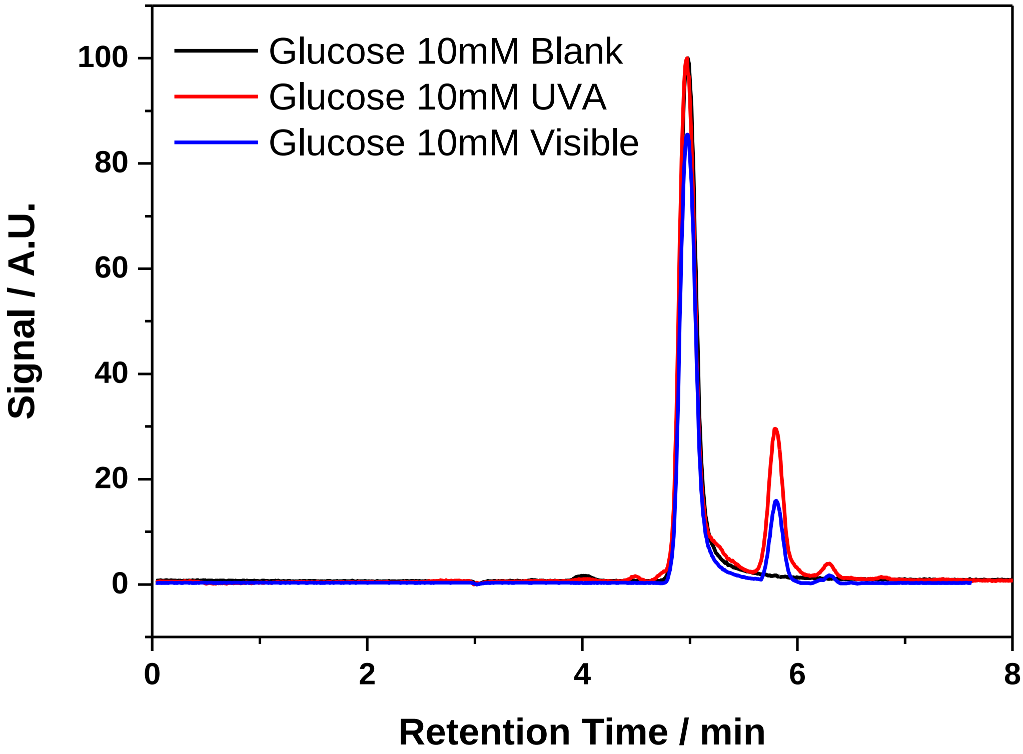

Figure S.5 Total Ion Chromatogram for the reaction samples of the 10mM blank glucose solution (black) and after exposure to the UVA lamp (red) and to the Xe lamp (blue) after 4 hours of reaction.

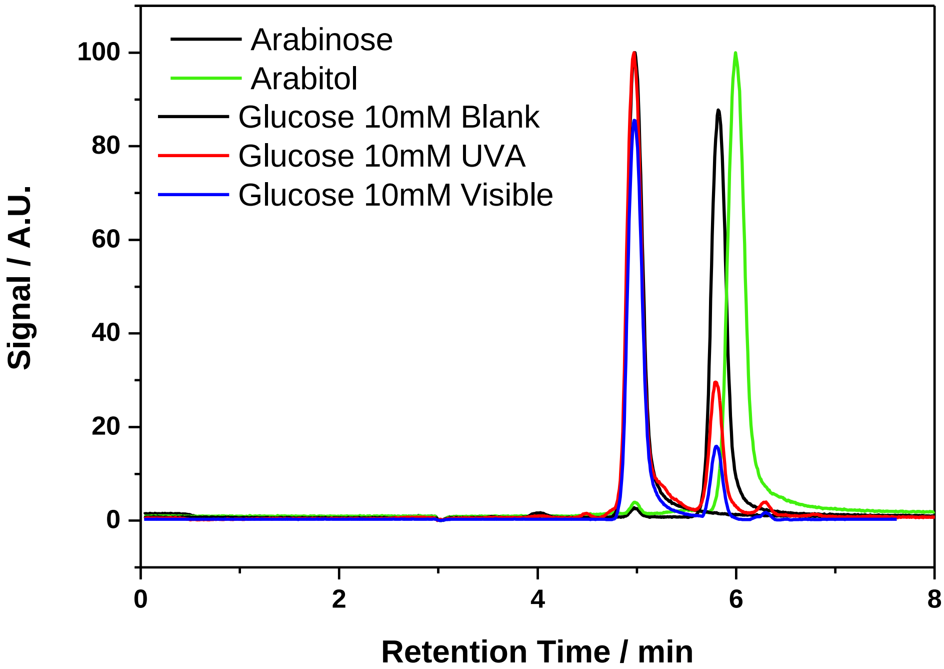

Figure S.6 Total Ion Chromatogram for the reaction samples of the 10mM glucose solution with the UVA lamp (red) and the Xe lamp (blue) after 4 hours of reaction. Additionally, the arabinose (black) and arabitol (green) standards are displayed. It can also be seen how in both cases traces of glucose are still visible in both chromatograms.

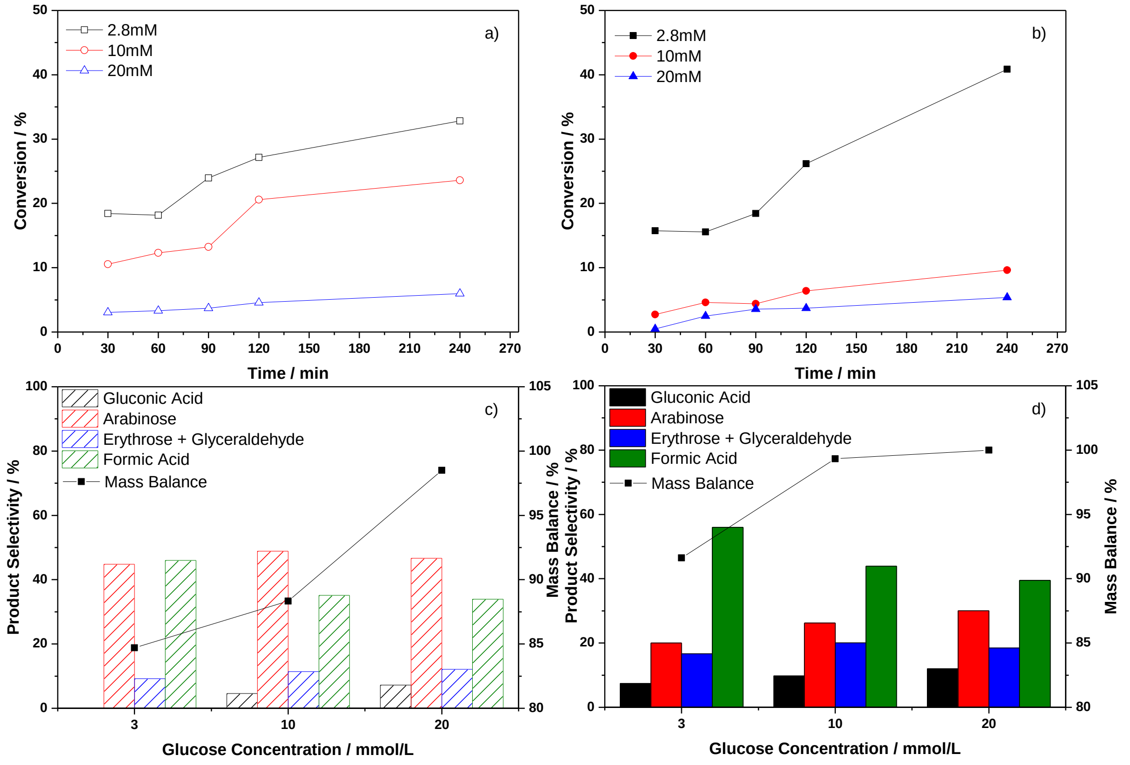

Figure S.8 Glucose oxidation results over TiO_2 blank (P25 Evonik) with different catalyst to substrate ratios: a) glucose conversion under UV light, b) glucose conversion under visible light, c) product selectivities and mass balance data under UV light, d) product selectivities and mass balance data under visible light.

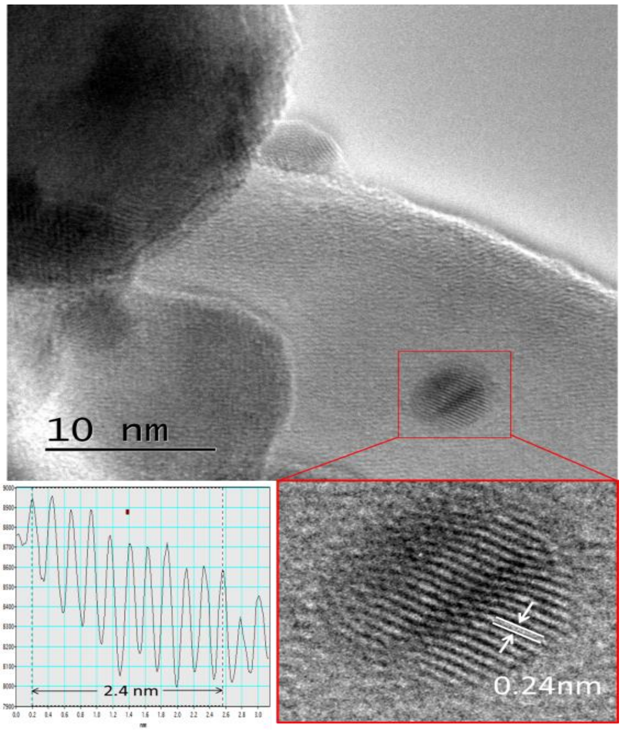

Figure S.9 HRTEM analysis of 0.5% $AgTiO_2$ showing Ag particles of *ca* 4.5nm. Bottom right: expanded image of Ag nanoparticle showing interplanar distances of 0.24 nm, Bottom left: Profile fit for 10 lattice planes.

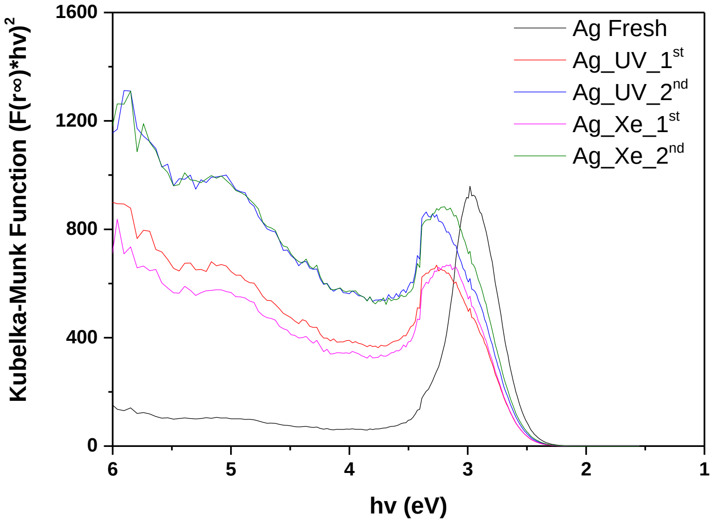

Figure S.10 Solid UV-Vis spectra of the 1 wt% Ag/TiO_2 catalyst after multiple reuses under visible and UVA light. The redshift of the plasmonic resonance peak can be observed upon multiple reuses. The shift was observed under visible and UVA light.

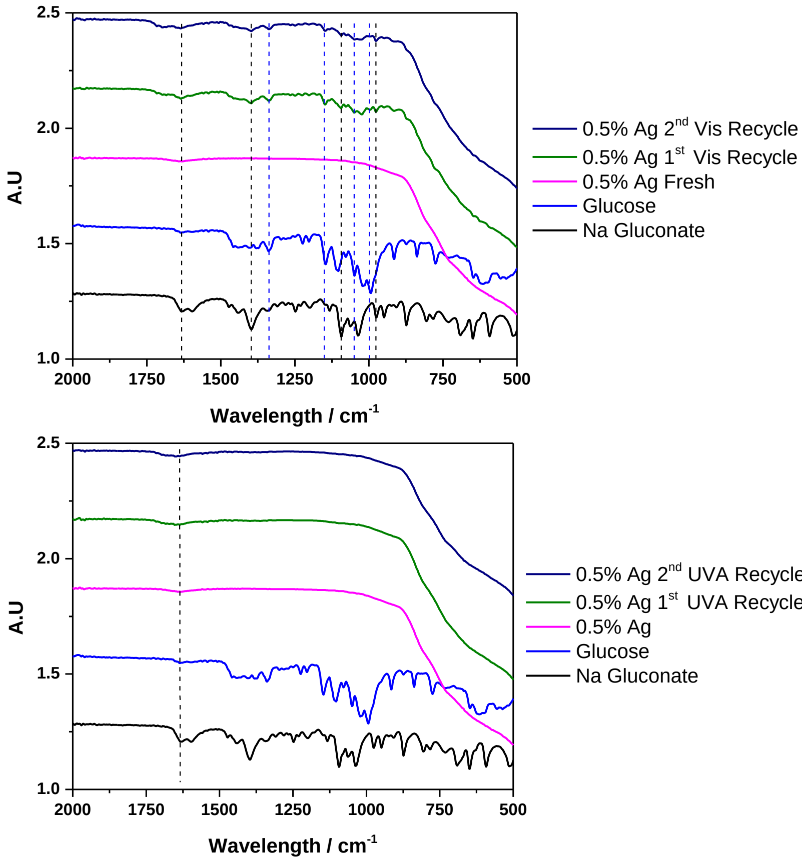

Figure S.11 Full data set for the FT-IR analysis of the 0.5 wt% Ag/TiO_2 catalyst before and after multiple reaction cycles under visible (top) and UVA light (bottom). Comparison with glucose and gluconate. Catalyst recycled twice, washed with ethanol and water and dried overnight under vacuum.

Table S1. Glucose and gluconic acid peak positions for Figure S.1

Catalyst	Glucose (blue) (cm^{-1})	Gluconic acid (black) (cm^{-1})
Ag 0.5 Vis 1st	915, 995, 1050, 1149, 1340, 1460	1095, 1202, 1401, 1636
Ag 0.5 Vis 2nd	915, 995, 1050, 1149, 1340, 1460	1095, 1202, 1401, 1636
Ag 0.5 UVA 1st	-	1636
Ag 0.5 UVA 2nd	-	

Table S2. Peak assignments for glucose and gluconic acid in Figure S.1

Glucose (blue)[1]		Gluconic acid (black)[2]	
IR (cm^{-1})	assignment	IR (cm^{-1})	assignment
1460	δCH_2+ δOCH+ δCCH	1636	$vCOO^-$ antisym
1340	δCCH+ δOCH	1401	$vCOO^-$ sym
1149	vCO+ vCC	1202	vCO + δCOO
1050	vCO+ vCC	1095	vCO + δCOO
995	vCO+ vCC		
915	vCO+ vCCH+ v_{as}ring of pyranose		

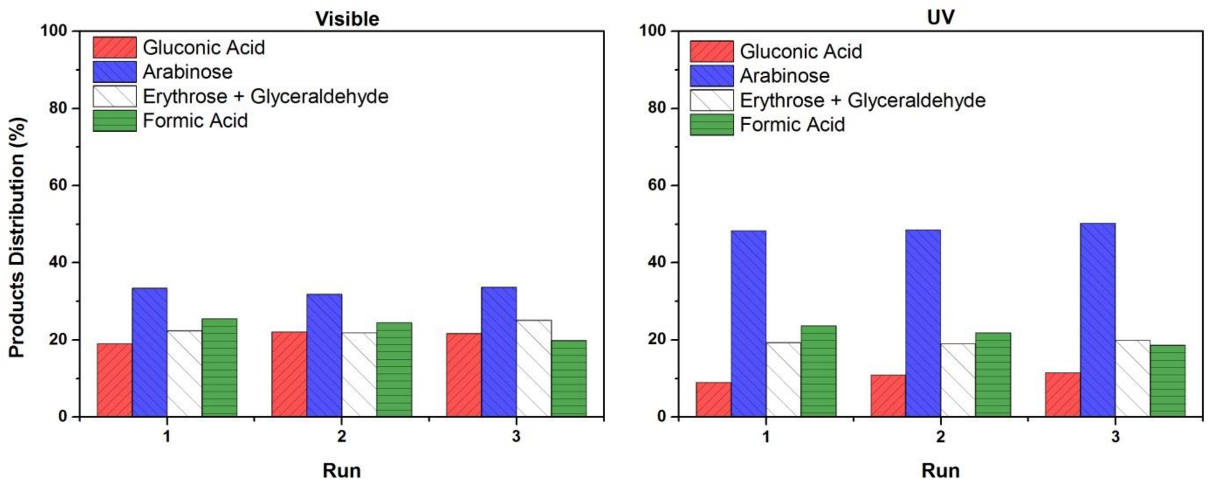

Figure S.12. Selectivity data for the recycling of the 1 wt% Ag/TiO_2 under visible (left) and UV light (right). 50/50 v/v MeCN/H_2O,14mg catalyst, 20mM glucose stock solution.

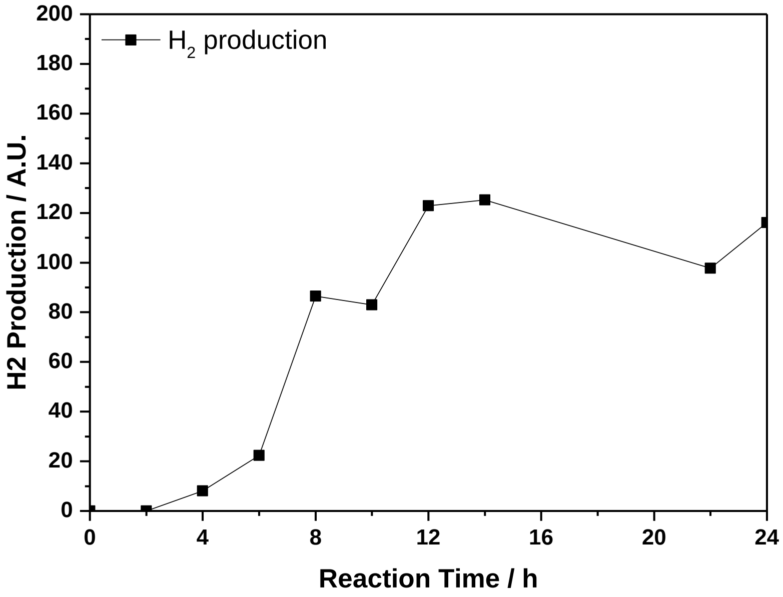

Figure S.13 Qualitative determination of the H_2 evolution for the 0.5 wt% Ag/TiO_2 sample over 24 hours. 50/50 v/v MeCN/H_2O, 14mg catalyst, 20mM glucose stock solution, visible light (λ >420 nm)

Solid UV-Vis Measurements

The graphs on the DR UV-Vis reflectance report the Kubelka-Munk function in the so-called Tauc plot.[3]

With this annotation, the equation which is defined by two parameters, the adsorption (K) and the scattering coefficient (S).

Eqn. 1 $$F(R_\infty) = \frac{(1-R_\infty)^2}{2R_\infty} = \frac{K}{S}$$

These two parameters are function of the particle size of the sample and the thickness of the layer to be analysed, and was originally applied for the analysis of paints on surfaces. This model assumes

that the sample layer can be divided in a large number of elementary layers which have boundaries parallel to the ones of the complete layer and have identical optical properties.[4]
By applying equation the second equation to the Kubelka-Munk, we obtain the following expression:

Eqn. 2 $$F(r_\infty) = \frac{(1-r_\infty)^2}{2r_\infty} = \frac{K}{S}$$

Due to the difficulty in measuring R_∞, defined as the reflectance of an infinite thick layer, another variable is needed, r_∞ defined as follows:

Eqn. 3 $$r_\infty = \frac{r_\infty(sample)}{r_\infty(standard\ material)}$$

Typically, $BaSO_4$ is used as a standard material as it has almost ideal reflecting properties across a wide wavelength range.[5] In fact, several manufacturers, apply a coating of this material inside the integrating sphere to insure a high signal to noise ratio to the detector to obtain higher sensitivity. The typical sigmoid shape of the reflectance is due to the absorption of the material at wavelengths around 400 nm, which causes the reflectance (R) to drop and have a constant value at wavelengths lower than 380 nm. In this work, we used $BaSO_4$ as a reference material to record the absorption profiles of several materials. But in some cases, when the TiO_2 was treated and it was necessary to distinguish between the modification of the support in addition to the presence of metal nanoparticles on the surface, different relationship were used to obtain information on the bandgap positioning and to evaluate the LSPR band position, therefore, sometimes the blank or untreated TiO_2 reflectance spectra was used as reference instead of the $BaSO_4$ blank. It is possible to determine the bandgap of solids using several arrays of equations, based on different assumptions. A good recent review on the application of different equations was published by Lopez and Gomez.[3]
In this study, the Kubelka-Munk function was modified multiplying the $F(R_\infty)$ by hv to obtain the energy of the bandgap in eV and by using a coefficient (n) associated with the specific transition considered. Therefore, the equation can be rewritten as follows identifying α as the extinction coefficient:

Eqn. 4 $$\alpha(h\nu) = (F(r_\infty) \cdot h\nu)^n$$

The coefficient is reported to be n=1/2 for direct allowed transition, and n=2 for indirect allowed transition. The so-called Tauc plot is the result of the relationship between Eqn. 4 and hv.[6]

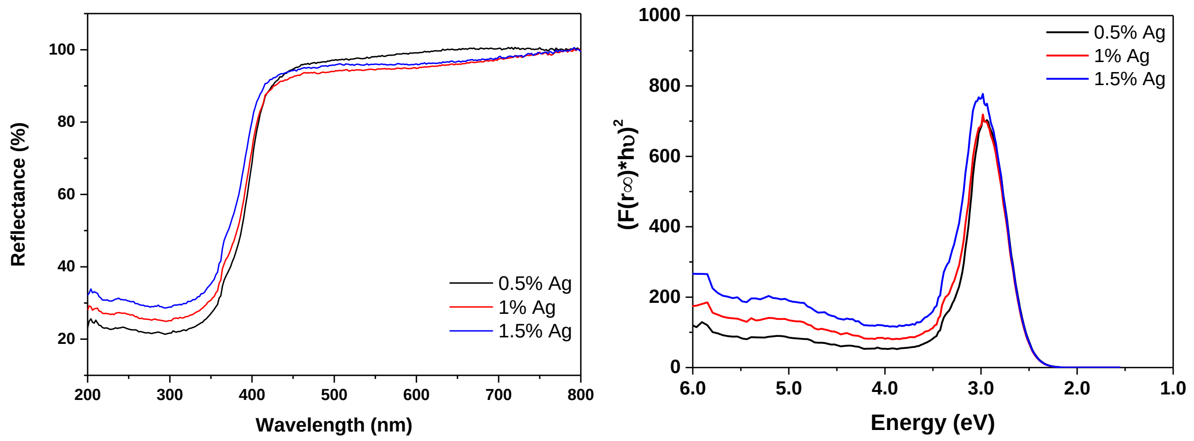

Figure S.14 UV-Vis absorption profile (left) for Ag/TiO_2 supported catalyst with different metal loadings and the corresponding Tauc plot (right) in which only the LSPR peak can be observed.

From Figure S.14 it is clear how the amount of information obtainable from the simple UV-Vis spectrum is not sufficient to assess the presence of metal nanoparticles on the surface of the TiO_2 especially if their absorption happens to be in the region where the semiconductor absorbs light. It is necessary to de-convolute the spectra, and the Tauc plot obtained using n=2 is displayed in Figure S.14 (right). The presence of the metal on the surface of the semiconductor is now clear, and additionally, it is possible to examine the nature of the nanoparticles (mono, bimetallic, core-shell) according to the peak shape and positioning.

[1] M. Ibrahim, M. Alaam, H. El-Haes, A. F. Jalbout, A. d. Leon, *Eclética Química* **2006**, *31*, 15-21.
[2] H.-A. Tajmir-Riahi, J. T. Agbebavi, *Carbohydrate Research* **1993**, *241*, 25-35.
[3] R. López, R. Gómez, *Journal of Sol-Gel Science and Technology* **2012**, *61*, 1-7.
[4] P. a. M. Kubelka, F., *Z. Tech. Phys* **1931**, *12*, (593-601).
[5] G. Kortüm, W. Braun, G. Herzog, *Angewandte Chemie International Edition in English* **1963**, *2*, 333-341.
[6] J. Tauc, R. Grigorovici, A. Vancu, *physica status solidi (b)* **1966**, *15*, 627-637.
